# Changes in facial surface temperature of laying hens under different thermal conditions

**DOI:** 10.5713/ab.20.0647

**Published:** 2020-12-01

**Authors:** Na Yeon Kim, Seong Jin Kim, Mirae Oh, Se Young Jang, Sang Ho Moon

**Affiliations:** 1 Department of Bio-Convergence Science, College of Biomedical and Health science, Konkuk University, Chungju 27478, Korea; 2 National Institute of Animal Science, RDA, Sunghwan 31000, Korea; 3 Institute of Livestock Environmental Management, Sejong 30127, Korea

**Keywords:** Laying Hens, Infrared Thermography, Surface Temperature, Heat Stress, Precision Poultry Farming

## Abstract

**Objective:**

The purpose of this study was to identify through infrared thermal imaging technology the facial surface temperature (FST) of laying hens in response to the variations in their thermal environment, and to identify the regional differences in FST to determine the most stable and reliable facial regions for monitoring of thermoregulatory status in chickens.

**Methods:**

Thirty Hy-Line Brown hens (25-week-old) were sequentially exposed to three different thermal conditions; optimal (OT, 22°C±2°C), low (LT, 10°C±4°C), and high temperature (HT, 30°C±2°C). The mean values of FST in five facial regions including around the eyes, earlobes, wattles, beak and nose, and comb were recorded through infrared thermography. The maximum FST (MFST) was also identified among the five face-selective regions, and its relationship with temperature-humidity index (THI) was established to identify the range of MFST in response to the variations in their thermal environment.

**Results:**

Hens exposed to OT condition at 15:00 displayed a higher temperature at wattles and around the eyes compared to other regions (p<0.001). However, under LT condition at 05:00 to 08:00, around the eyes surface temperature showed the highest value (p<0.01). In HT, wattles temperature tended to show the highest temperature over almost time intervals. Main distribution regions of MFST were wattles (63.3%) and around the eyes (16.7%) in OT, around the eyes (50%) in LT, and wattles (62.2%) and comb (18.3%) in HT. The regression equation between MFST and THI was estimated as MFST = 35.37+ 0.2383×THI (R^2^ = 0.44; p<0.001).

**Conclusion:**

The FST and the frequency of MFST in each facial region of laying hens responded sensitively to the variations in the thermal environment. The findings of this experiment provide useful information about the effect of the thermal conditions on the specific facial regions, thus offering an opportunity to stress and welfare assessment in poultry research and industry.

## INTRODUCTION

Recently, global environmental temperature increase due to global warming and climate change are getting worse every year, and livestock cannot be free from the effects. Heat stress occurs when the net heat dissipation flowing from the body to its surrounding environment is less than the amount of heat energy produced by the body [1], and it causes serious losses in animal welfare and productivity. Heat stress reduces the feed intake, body weight, production efficiency and metabolic rate of animals [2,3]. In broilers, heat stress increased pale, soft and exudative meat, drip loss and lactate content in the muscle, whereas it reduced protein synthesis in the breast muscle, caused oxidative damage to skeletal muscle and had a harmful effect on meat color and pH value [4,5]. Heat stress not only caused a reduction in feed intake, body weight, dietary digestibility, dietary calcium, serum protein and calcium content but also caused reproductive problems, poor egg quality, impaired skeletal integrity, poor egg mass and poor egg production in laying hens [6,7]. Poor performance under heat stress is accompanied by not only drop in the growth performance but also increasing mortality of broilers [8].

The heat stress levels animals feel vary according to environmental factors, such as ambient temperature, relative humidity, solar radiation and wind speed, and animal management strategies, such as feed, water, environment modifications and handling changes. In addition, animal characteristics such as species, color, sex, age, temperament, previous exposure, body condition and current health could also affect the extent of heal stress [3,9]. Therefore, the heat stress from which animals suffer can be diagnosed accurately when a variety of measurement methods are combined to reflect animal-based criteria in addition to measuring environmental factors.

Ambient temperature is a crucial climatic factors that has a strong effect on maintaining the body temperature within the normothermic ranges in domestic fowls; therefore, their body temperature monitoring will provide useful information in regard to the intensity of thermal stressors on their thermoregulatory status [10,11]. The conventional methods directly measure the body temperature, and thus are associated with invasiveness, and being time consuming and labor-intensive. Therefore, conventional methods would potentially result in measurement errors. In response to these shortcomings, infrared thermal imaging technology offers a fast, highly precise, non-invasive, and non-contact method for measuring the skin surface temperature [12]. Furthermore, this technology enables to continuously collect the data during the stress period, which eliminates the confounding effects associated with frequent capturing of animal and sampling [13].

A strong correlation has been reported between body temperature and facial surface temperature (FST) in chicken [11]; therefore, the variations in environmental temperature could rapidly be manifested in skin surface temperature, which makes it an important parameter in monitoring the comfort or thermal stress of chickens using infrared thermography [14–16]. For skin temperature measurement using infrared thermography in poultry studies, bare areas such as the eye, comb and wattle, earlobes, or nose are the potential zones for detection of temperature differences as thermal stressor intensity changes. Herborn et al [13] identified temperature differences in the skin surface of hens as thermal intensity shifted from a mild to a more severe acute stress. However, to our knowledge less is known about the contributions of different facial surface sites of laying hens when exposed to thermally comfortable or stressful conditions. Therefore, this study was aimed at identifying the regional differences in the FST of laying hens through infrared thermography technology in response to the thermal variations in the environment.

## MATERIALS AND METHODS

### Animals and management

This experiment was implemented under the approval of the Institutional Animal Care and Use Committee of Konkuk University (IACUC No. KU 19003). Thirty 25-week-old Hy-Line Brown laying hens were placed in a 3×7 m experimental room. Three hens were placed in a battery cage with a stocking density of 420 cm^2^/hen, so whole hens were assigned to ten cages. Ambient temperature and relative humidity were maintained using a ventilation fan and a thermal insulation facility. The photoperiod was 16 L:8 D with lights on from 05:20 to 21:20 h, and the lighting was kept above 10 lux during the bright period. The feed for the early laying period (20 to 40 weeks of age) was given 150 g twice a day, morning (07:00 h) and afternoon (14:00 h). Freshwater was available freely through a nipple and feces were cleaned once a day.

Prior to data collection, hens were given a 15-d adaptation period to experimental conditions and stocking density (temperature = 22°C±2°C, mean±deviation; relative humidity = 44% – 62%). After adaptation period, the data were collected under optimal temperature (OT, 22°C± 2°C) for 3 days. The temperature was then switched to low temperature (LT, 10°C±4°C; mean±deviation) and hens were kept in LT for 7 days (4 days of adaptation + 3 days of data recording). Hens were given a buffer period of 22°C±2°C for 7 days. The temperature was then switched to high temperature (HT, 30°C±2°C; mean±deviation) for a period of 7 days (4 days for adaptation and 3 days for data recording).

### Facial surface temperature measurement

The FST data (n = 30) were collected at six intervals: 05:00 h, 08:00 h, 12:00 h, 15:00 h, 18:00 h, and 21:00 h during three collection days using infrared thermal camera (CX320; COX Co., Daejeon, Korea). Ambient temperature and relative humidity were also recorded five times a day, according to the infrared thermal imaging sampling time, using mercury thermometer YY-11 (Dong-myeong, Seoul, Korea) and the psychrometer TM0081 (TQC, Capelle aan den IJssel, Netherlands). The calibration of the thermometer and the psychrometer were guaranteed by the professional experts. The following equation was used for calculation of temperature-humidity index (THI) specified for laying hens [17]:

THI=0.40×wet-bulb temperature (°C)+0.60×dry-bulb temperature (°C)

**Figure 1:**
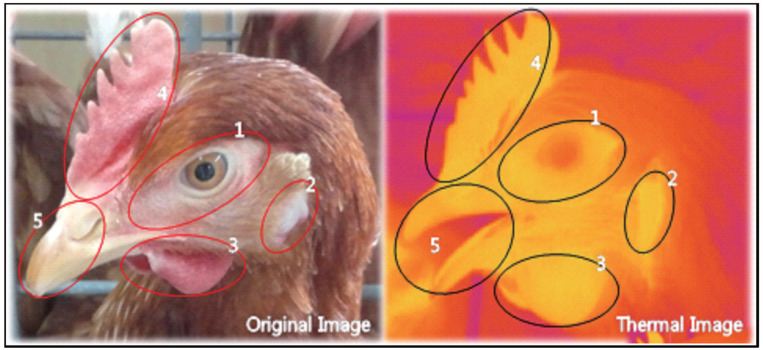
Illustration of laying hen’s face displaying the face-selective regions. Left picture is a visible light image and right picture is an infrared thermographic image. The red area is illustrative of low temperature and the yellow area is illustrative of high temperature. The elliptical zones show face-selective regions. Region 1 = around the eyes; region 2 = earlobes; region 3 = wattles; region 4 = comb; region 5 = beak and nose.

Five distinct facial areas – around the eyes, earlobes, wattle, comb and beak and nose – were selected for FST measurements (Figure 1). Three images were taken at each distinct facial area of each individual on each time interval, and the average temperature was determined using Thermal Imaging Analyzer software ver. A.8 (COX Co., Korea). The maximum FST (MFST) was defined as the maximum temperature among the five facial regions in individual hens. The individual MSFT values at each time point (n = 30) were collected and the mean value was calculated. The proportion of each facial region to MFST was also determined at each collection time for the three experimental group.

### Statistical analysis

The data of FST were compared among the five face-selective regions and those of MFST to identify the regional differences in FST. Also, the MFST values were compared among the three experimental groups. The proportion data of each facial region to MFST was compared among the five regions for each experimental group to detect the most stable and reliable facial regions for monitoring of thermoregulatory status in laying hens. These data were analyzed by using analysis of variance (ANOVA) of the general linear model procedure in SAS ver. 9.1 (SAS Institute, Cary, NC, USA). When a statistically significant difference was identified, Duncan’s multiple range test was used to detect statistical significance (p<0.05). The correlation and regression analysis between MFST and THI were also conducted to identify the range of FST of laying hens under different thermal environment. The correlation data were statistically analyzed by using Pearson’s product-moment correlation and the regression data were analyzed by using ANOVA of the simple linear regression analysis in R studio ver. 1.3.1073 (R studio, Boston, MA, USA).

## RESULTS

### Facial surface temperature

Changes in FST of the five specific regions under different thermal conditions are presented in Table 1. Under OT exposure, wattle and MFST temperatures at 05:00 h were 38.31°C and 38.58°C respectively, which were higher than that of earlobes, beak and nose (p<0.001), but no significant difference was detected with comb and around the eyes regions. At 08:00 h, the MFST was 39.30°C, which was significantly higher than those of wattles, comb, beak and nose (p<0.001), but the significance with around the eyes and earlobes was not observed. At 12:00 h, the MFST and wattles were 40.35°C and 40.32°C respectively, which were higher than that of beak and nose (p<0.001), but no significant difference was detected with around the eyes, earlobes and comb regions. At 15:00 h, MFST, wattles and around the eyes recorded the highest temperature (mean = 41.66°C) among the other regions (p<0.001). At 18:00 h, MFST and wattles temperatures were 39.2°C and 39.01°C respectively, which were higher than all regions except around the eyes (p<0.001). At 21:00 h, the MFST, around the eyes and wattles were 41.05°C, 40.65°C, and 40.27°C respectively, which were higher than that of earlobes (p<0.001), but no significant difference was detected with comb, beak and nose regions.

Under LT condition, the temperatures of MFST and around the eyes were 35.1°C and 34.8°C at 05:00 h, 37.7°C and 37.5°C at 08:00 h, respectively, which were the highest among all regions (p<0.001). At 12:00 h, MFST, wattles and around the eyes temperatures were 41.01°C, 40.7°C, and 40.1°C, respectively, showing significantly higher than the other areas (p< 0.001). At 15:00 h, the temperatures of MFST, around the eyes and wattle were 39.4°C, 38.72°C, and 38.68°C, respectively, which were the highest among the other regions (p< 0.001). At 18:00 h, MFST temperature was 37.6°C, which is significantly higher than other regions except for around the eyes (p<0.001). At 21:00 h, the temperature of MFST was 35.7°C, which is significantly higher than other regions except for around the eyes (p<0.01).

Hens exposed to HT displayed mean MFST and wattle temperatures of 39.8°C and 39.7°C at 05:00 h respectively, which were the highest value of all regions (p<0.001). At 08:00 h, the temperatures of MFST, wattle, comb, and around the eyes were 40.4°C, 40.2°C, 40.0°C, and 39.95°C, respectively, which were higher than the remaining regions (p<0.001). At 12:00 h, although MFST and wattle temperatures (45.41°C and 45.39°C) were comparable to comb, the other regions recorded a lower temperature (p<0.001). At 18:00 h, the MFST was 39.44°C, which was higher than that of around the eyes, earlobes, beak and nose (p<0.001), but no significant difference was detected with wattles and comb. At 21:00 h, MFST and wattles temperatures were 41.6°C and 41.52°C, which were higher than that of earlobe, beak and nose (p< 0.05), but no difference was recognized with those of comb and around the eyes.

### Maximum facial surface temperature

Changes in MFST distribution among the five facial regions in the laying hens exposed to optimal, low and high temperature are presented in Table 2. Compared with LT, HT-exposed hens recorded a significantly higher MFST at all collection times (p<0.001), and this difference was the highest at 21:00 h (41.61°C vs 35.68°C). Compared with OT, hens kept in HT condition exhibited higher MFST values from 5:00 h to 12:00 h (p<0.001). However, no significant difference existed between HT and OT at 15:00 h and 21:00 h. In general, hens that were exposed to OT exhibited values of MFST ranging from 38.58°C through 41.88°C, which were significantly higher than those obtained from LT-exposed hens in all time intervals, with the exception for 12:00 h (p<0.001) when LT group displayed a higher value (40.35°C vs 41.01°C).

The proportion of each facial region to MFST in laying hens kept under different thermal conditions is shown in Table 3. In OT group, MFST was more frequently detected at wattle (63.3%) and around the eyes (16.7%) regions (p< 0.001), together contributing to 80% MFST values. In LT, MFST was frequently spotted at around the eyes (50%; p< 0.01). Around the eyes and wattles regions together contributed to 77.8% MFST values. In HT-exposed hens, MFST values were spotted in high proportion at wattle (62.2%) and comb (18.3%) regions (p<0.001), together comprising 80.5% of MFST values.

**Figure 2:**
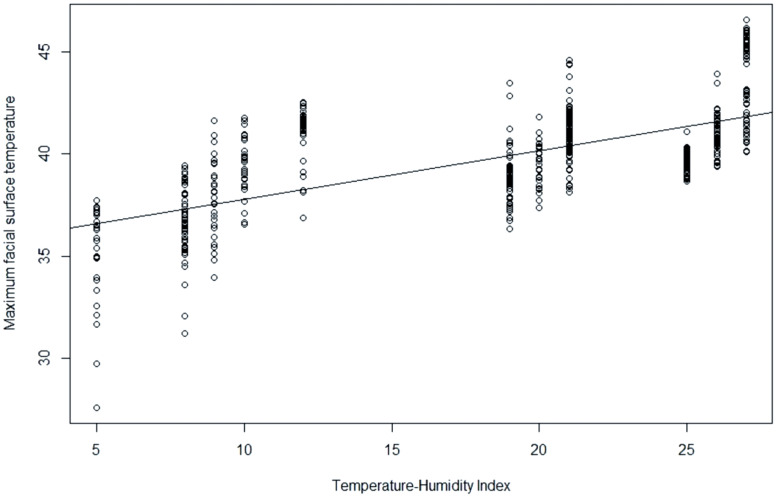
Scatter plot and regression model between temperature-humidity index (THI) and maximum facial surface temperature of laying hens (r = 0.67; R^2^ = 0.45; p<0.001). Data were expressed as means and were statistically analyzed with analysis of variance using simple linear regression analysis. Residual standard error was 1.949 on 538 degrees of freedom.

As illustrated in Figure 2, a significant correlation was observed between MFST and THI (r = 0.64, p<0.001). The regression equation between MFST and THI was estimated as MFST = 35.37+0.2383×THI (R^2^ = 0.44; p<0.001).

## DISCUSSION

One of the notable features of the FST is that it is highly affected by dynamic environmental temperature changes. The core temperature in hens is regulated within the range of about 40.6°C to 41.7°C [18] but in this experiment, 10.27°C was the maximum difference of the mean MFST between the ambient temperature group of 6°C and 32°C. In addition, the standard error of the mean (SEM) associated with MFST tended to be smaller with the elevation of ambient temperature; however, higher values for SEM were obtained as ambient temperature declined. For FST measurements, the maximum surface temperature is an important parameter to indirectly measure the core temperature of animals as it more closely resembles the core temperature (Table 1). MFST, the result of body heat exchange, does not rise above the core temperature if the homeostasis of warm-blooded animals is maintained. At the proper temperature (OT), MFST ranged from 38.58°C to 41.88°C, which is generally close to the body core temperature in hens. This association demonstrates that the FST measurements using the thermal imaging camera is a suitable method in reflecting normal body temperature in hens when the environmental temperature is appropriate. On the other hand, the MFST value of 45.41°C recorded at 12:00 h in HT-exposed hens substantially exceeded the normal core temperature range (40.6°C to 41.7°C), which could possibly be explained by the inefficient body heat dissipation in laying hens when kept in heat stress situations. The ambient temperature at this time interval (12:00) was 32°C, which was the highest among the values recorded during HT experiment. A study with laying hens revealed that when THI increased from 25 to 29, hens experienced critical heat stress as evidenced by reductions in feed consumption and egg production, and increase in mortality rate [19]. This might provide an indication that in the current experiment, HT-exposed hens at 12:00 experienced a stressful situation as the mean THI was 27.

Environmental temperature and relative humidity are the major factors that determine body surface heat exchange, and FST measurement using infrared thermography is believed to reflect the rapid changes in body surface temperature. However, it is not enough to consider only ambient temperature and humidity as determinants in order to more sensitively detect changes in FST of laying hens. This is indicated by the fact that FST was measured high even though ambient temperature was not high, such as LT at 12:00 h or OT at 15:00 to 21:00 h. Heat stress results from an imbalance in the homeostasis of the animals, and the level of heat stress that an animal experiences is related to three main factors: the weather conditions, animal characteristics, as well as management protocols used [3,9]. Temperature, humidity, wind speed and solar radiation are important weather components involved in the extent of heat stress as animal experiences [3]. Therefore, several equations have been developed to help summarize these factors into a single usable number [20]. In laying hens, additional parameters such as laying age, the performance of laying, laying time, feeding time, active time, the diurnal rhythm of core temperature as well as species, color, sex, age, temperament, previous exposure, body condition, current health [9] should be considered for animal susceptibility. In addition, since core body temperature (CBT) is the body temperature that the animal maintains for body homeostasis, CBT measurements in the future studies and comparison with the CBT value with the body surface temperature value can specifically support the effect on the sensitivity of the animal [21]. To this end, further studies should be carried out to identify a correlation between each factor for hens’ susceptibility and heat loss and production, and this effort will help detect more accurate and sensitive FST in laying hens.

Laying hen is a small animal, therefore, all regions of the face could easily be included in one photo, which results in MFST that incorporates the surface temperature values of the regions. However, infrared thermography can produce errors depending on the measurement distance or angle. Therefore, in order to minimize these errors, it is necessary to identify which facial region in the laying hen is the most reliable to record the highest distribution of MFST. Wattle surface temperature was the most stable part which showed the highest level of heat production at constant values under any thermal variations. However, the MFST values were frequently identified at around the eyes region in LT-exposed hens. Therefore, wattles and around the eyes could be the most reliable regions for FST measurement in laying hens. However, depending on the environmental temperature, wattles and around the eyes, around the eyes and wattles, wattles could be more reliable at optimal, low, and high temperatures, respectively.

In the present experiment, the poor correlation between MFST and THI and the coefficient of determination of the regression equation might be explained by the measurement method through infrared thermography. Since thermal images were measured manually by the attendant entering the experiment room at each measurement time, the laying hens could be affected by human access. In order to minimize interference due to human approach, the measurement distance of the thermal imaging camera was set at 1.5 m. In this experiment, however, the target subject for thermal image recording was the head of a small animal that is a very small area. Therefore, it is judged that the accurate temperature could not be sensitively detected because the size of the measurement object was small, and the distance was far. In actual farm situations, more accurate FST data will be obtained if the real-time automatic measurement systems could be installed at appropriate spots such as feed bins, water supply nipples, and egg laying boxes. In general, hens have a better ability to cope with low than high temperatures [18], and there is an increasing burden of long-term exposure to high temperatures in the summer, rather than the risk of exposure to low temperatures when managed in a henhouse. When the environmental temperature exceeds 30°C, the difficulty of body heat dissipation given to thick feathered hens increases, leading to high temperature stress. In this situation, the FST of laying hens has the advantage of being closer to the CBT [16] and being easier to access and measure than the surface temperature of other areas such as wings, legs, breast, and back.

## CONCLUSION

Overall, body surface temperature measurements using infrared thermal imaging cameras can be applied to a large number of subjects in a short time, significantly reducing the time and effort acting as a burden on managers who measure body temperature, and there is also no stress on the livestock, which is the measurement target. The findings of this study enabled a better understanding of the temperature differences in the facial regions of hens in response to the ambient temperature fluctuations. Such data could be viewed as potential markers for identification of thermal stress intensity, and thus a useful way to improve the welfare of hens during the high-temperature periods.

## Figures and Tables

**Table 1:** Changes in facial surface temperature in different regions of the laying hens under different thermal conditions (n = 30)

Collection time	Facial region	OT (22°C±2°C)			LT (10°C±4°C)			HT (30°C±2°C)		
		
		Mean	SEM p-value	AT RH (THI)	Mean	SEM p-value	AT RH (THI)	Mean	SEM p-value	AT RH (THI)
05:00	AE	37.65^ab^	0.46	20°C	34.77^a^	1.00	6°C	39.02^b^	0.34	28°C
	EL	37.00^b^	<0.001	60% (19)	32.04^b^	<0.001	62% (5)	38.30^c^	<0.001	61% (25)
	WT	38.31^a^			32.47^b^			39.67^a^		
	CB	37.74^ab^			25.54^c^			38.97^bc^		
	BN	35.61^c^			23.22^d^			38.62^c^		
	MFST	38.58^a^			35.14^a^			39.80^a^		
08:00	AE	38.90^ab^	0.55	22°C	37.47^a^	0.93	10.5°C	39.95^a^	0.35	29°C
	EL	38.39^abc^	<0.001	57% (20)	34.47^b^	<0.001	58% (9)	38.79^b^	<0.001	55% (26)
	WT	38.07^bc^			35.19^b^			40.16^a^		
	CB	37.75^b^			32.55^c^			40.00^a^		
	BN	34.99^d^			26.02^d^			39.18^b^		
	MFST	39.30^a^			37.66^a^			40.39^a^		
12:00	AE	39.70^ab^	0.41	23°C	40.11^a^	0.52	14°C	45.05^b^	0.26	32°C
	EL	38.90^ab^	<0.001	51% (21)	38.58^b^	<0.001	53% (12)	44.52^c^	<0.001	50% (27)
	WT	40.32^a^			40.65^a^			45.39^a^		
	CB	39.58^ab^			38.49^b^			45.11^ab^		
	BN	38.23^b^			37.62^b^			44.87^b^		
	MFST	40.35^a^			41.01^a^			45.41^a^		
15:00	AE	41.27^a^	0.54	24°C	38.72^a^	0.78	13°C	41.25	0.22	31°C
	EL	40.51^b^	<0.001	46% (21)	37.19^b^	<0.001	47% (10)	41.01	0.112	47% (26)
	WT	41.83^a^			38.68^a^			41.52		
	CB	40.41^b^			35.41^c^			41.23		
	BN	37.91^c^			31.41^d^			41.07		
	MFST	41.88^a^			39.40^a^			41.60		
18:00	AE	38.64^ab^	0.45	22°C	37.18^ab^	0.79	10°C	39.01^b^	0.30	29°C
	EL	37.43^cd^	<0.001	45% (19)	35.41^c^	<0.001	45% (8)	38.38^c^	<0.001	44% (25)
	WT	39.01^a^			36.00^bc^			39.33^ab^		
	CB	37.72^bc^			31.80^d^			39.25^ab^		
	BN	36.67^d^			29.90^e^			38.51^c^		
	MFST	39.20^a^			37.63^a^			39.44^a^		
21:00	AE	40.65^a^	0.39	23°C	35.20^ab^	0.87	9°C	41.27^ab^	0.27	30°C
	EL	38.99^b^	<0.001	62% (21)	33.37^c^	0.004	59% (8)	40.80^b^	0.011	60% (27)
	WT	40.27^a^			33.59^bc^			41.52^a^		
	CB	39.70^ab^			26.90^d^			41.25^ab^		
	BN	39.64^ab^			27.67^d^			40.77^b^		
	MFST	41.05^a^			35.68^a^			41.61^a^		

AT and RH collected for 3 days in each temperature treatment are average data of the experimental period (3 days in each treatment).

OT, optimal temperature; LT, low temperature; HT, high temperature; SEM, standard error of the mean; AT, ambient temperature (°C); RH, relative humidity (%); THI, temperature-humidity index; AE, around the eyes; EL, earlobes; WT, wattles; CB, comb; BN, beak and nose; MFST, maximum facial surface temperature;

a–e Means within a column without a common superscript differ (p<0.05).

**Table 2:** Changes in the maximum facial surface temperature among the 5 facial regions of the laying hens under different thermal conditions (n = 30)

Collection time	Mean			SEM	p-value

	OT (22°C±2°C)	LT (10°C±4°C)	HT (30°C±2°C)		
05:00 h	38.58^b^	35.14^c^	39.80^a^	0.90	<0.001
08:00 h	39.30^b^	37.66^c^	40.39^a^	0.68	<0.001
12:00 h	40.35^c^	41.01^b^	45.41^a^	0.96	<0.001
15:00 h	41.88^a^	39.40^b^	41.60^a^	0.67	<0.001
18:00 h	39.20^a^	37.63^b^	39.44^a^	0.57	<0.001
21:00 h	41.05^a^	35.68^b^	41.61^a^	1.07	<0.001

OT, optimal temperature; LT, low temperature; HT, high temperature; SEM, standard error of the mean.

a–c Means within a row without a common superscript differ (p<0.05).

**Table 3:** Proportion of each facial region to maximum facial surface temperature in laying hens under different thermal conditions (n = 30)

Thermal condition	Facial region					SEM	p-value

	AE	EL	WT	CB	BN		
	---------------------------------------------------------------- % ---------------------------------------------------------						
OT (22°C±2°C)	16.7^b^	8.9^c^	63.3^a^	9.4^c^	1.7^d^	2.23	<0.001
LT (10°C±4°C)	50.0^a^	13.9^bc^	27.8^b^	7.8^bc^	0.6^c^	1.98	0.002
HT (30°C±2°C)	7.2c	8.3^c^	62.2^a^	18.3^b^	3.9^c^	2.20	<0.001

AE, around the eyes; EL, earlobes; WT, wattles; CB, comb; BN, beak and nose; SEM, standard error of the mean; OT, optimal temperature; LT, low temperature; HT, high temperature.

a–d Means within a row without a common superscript differ (p<0.05).
